# Seasonal Food Availability, Preservation Practices, and Dietary Diversity in Children Under 5 in Western Kenya

**DOI:** 10.1002/puh2.70200

**Published:** 2026-02-18

**Authors:** Gladwel Kuya Sikoyo, Judith Mangeni, Diana Menya, Oscar Kambona, Antony Ochung, Fanuel Kawaka, George Ayodo

**Affiliations:** ^1^ Department of Public and Community Health Jaramogi Oginga Odinga University of Science and Technology Bondo Kenya; ^2^ Department of Epidemiology and Medical Statistics Moi University Eldoret Kenya; ^3^ Department of Nutrition Siaya County Government Siaya Kenya; ^4^ Department of Biological Sciences Jaramogi Oginga Odinga University of Science and Technology Bondo Kenya; ^5^ Center for Community Health and Wellbeing Jaramogi Oginga Odinga University of Science and Technology Bondo Kenya

**Keywords:** anthropometric, indicators, malnutrition, socioeconomic, stunting

## Abstract

Malnutrition remains a leading cause of morbidity and mortality among children under 5 years worldwide. In Kenya, the situation is further aggravated by climate change, which disrupts agricultural productivity and limits access to nutrient‑rich foods. This study explored the availability, seasonal patterns, and preservation of local foods as strategies for improving minimum dietary diversity (MDD) among children under 5 in Siaya County, Kenya. A cross‑sectional study employing both descriptive and inferential statistical analyses was conducted among children aged 0–60 months, their caregivers, and local food vendors. Anthropometrics were assessed using World Health Organization (WHO) standards, and questionnaires captured demographic, dietary, and seasonal food availability information. Statistical analyses included Chi‑square tests and logistic regression. Results showed low consumption of nutrient‑rich foods such as legumes/nuts, eggs, and flesh foods, with only 19% (*n* = 21) of children meeting MDD. Children who met MDD consumed significantly more legumes/nuts, eggs, and flesh foods (*p* < 0.001). They also showed that children had an average age of 16.7 months; 58% (*n* = 64) were female, and 71.2% (*n* = 78) were between 6 and 23 months old. This study highlights dietary vulnerability among children aged 6–23 months and emphasizes the need to strengthen dietary diversity and food preservation practices.

## Graphical abstract

Seasonal food availability and limited preservation practices constrain dietary diversity among children under 5 in Siaya County, Kenya. Only 19% of children met the WHO minimum dietary diversity threshold, with low consumption of eggs, legumes, and animal‐source foods highlighting the need for climate‐resilient nutrition strategies.

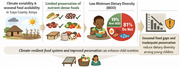

## 1 Introduction

Malnutrition remains a critical global health challenge, affecting more than 820 million people worldwide and contributing substantially to morbidity and mortality among children under 5 years. In 2020, approximately 1.3 billion individuals experienced nutritional insecurity [1, 2]. Undernutrition remains a major cause of disease burden, particularly in low‐ and middle‐income countries, where children under 5 are disproportionately affected. [3]. In East Africa, the burden of child malnutrition is extremely high, with chronic malnutrition affecting approximately 30.6% of children under 5 compared to the global average of 22% [4]. Wasting, an indicator of acute malnutrition, affects about 5% of children in the region [5]. Kenya continues to face a serious malnutrition crisis, with 18% of children under 5 classified as stunted and malnutrition contributing to nearly half of the approximately 70,000 annual deaths among children in this age group [6]. In Siaya County, the situation is more severe than national averages, with stunting at 19.2%, underweight at 7.0%, and wasting at 1.7% [6].

Climate change intensifies malnutrition in Sub‐Saharan Africa by disrupting food systems through altered rainfall patterns and increasingly frequent extreme weather events, such as droughts and floods [7]. These environmental shifts reduce the availability, diversity, and nutrient quality of food [8]. This shift increases risks of undernutrition and micronutrient deficiencies [3]. Minimum dietary diversity (MDD), a proxy indicator of diet quality, measures the proportion of children aged 6–23 months who consume foods from at least five of the eight World Health Organization (WHO)‐recommended food groups within a 24 h period [3]. Studies show that diverse diets incorporating locally available foods enhance resilience to climate variability [9]. In Siaya County, however, seasonal food shortages, limited food preservation methods, and climate variability limit households’ ability to consistently meet MDD requirements [10]. Existing interventions have focused primarily on increasing food quantity with limited attention to improving dietary quality and diversity, leaving micronutrient deficiencies largely unaddressed [11, 12].

Although indigenous foods and traditional preservation practices can improve dietary diversity, their seasonal availability and nutritional contribution remain insufficiently studied [13]. Moreover, few studies have examined how intra‐seasonal dietary diversity fluctuates in response to climate shocks or how integrated socioeconomic and environmental factors shape child nutrition outcomes in Siaya County [14, 15]. This study addresses key gaps by examining seasonal food availability, preservation methods, and dietary diversity among children under 5 in Siaya County. Understanding these factors is essential for designing contextual, climate‐resilient nutrition strategies aimed at improving MDD and enhancing child nutrition outcomes among children under 5 in Siaya County, western Kenya.

## 2 Materials and Methods

### 2.1 Study Site

**FIGURE 1:**
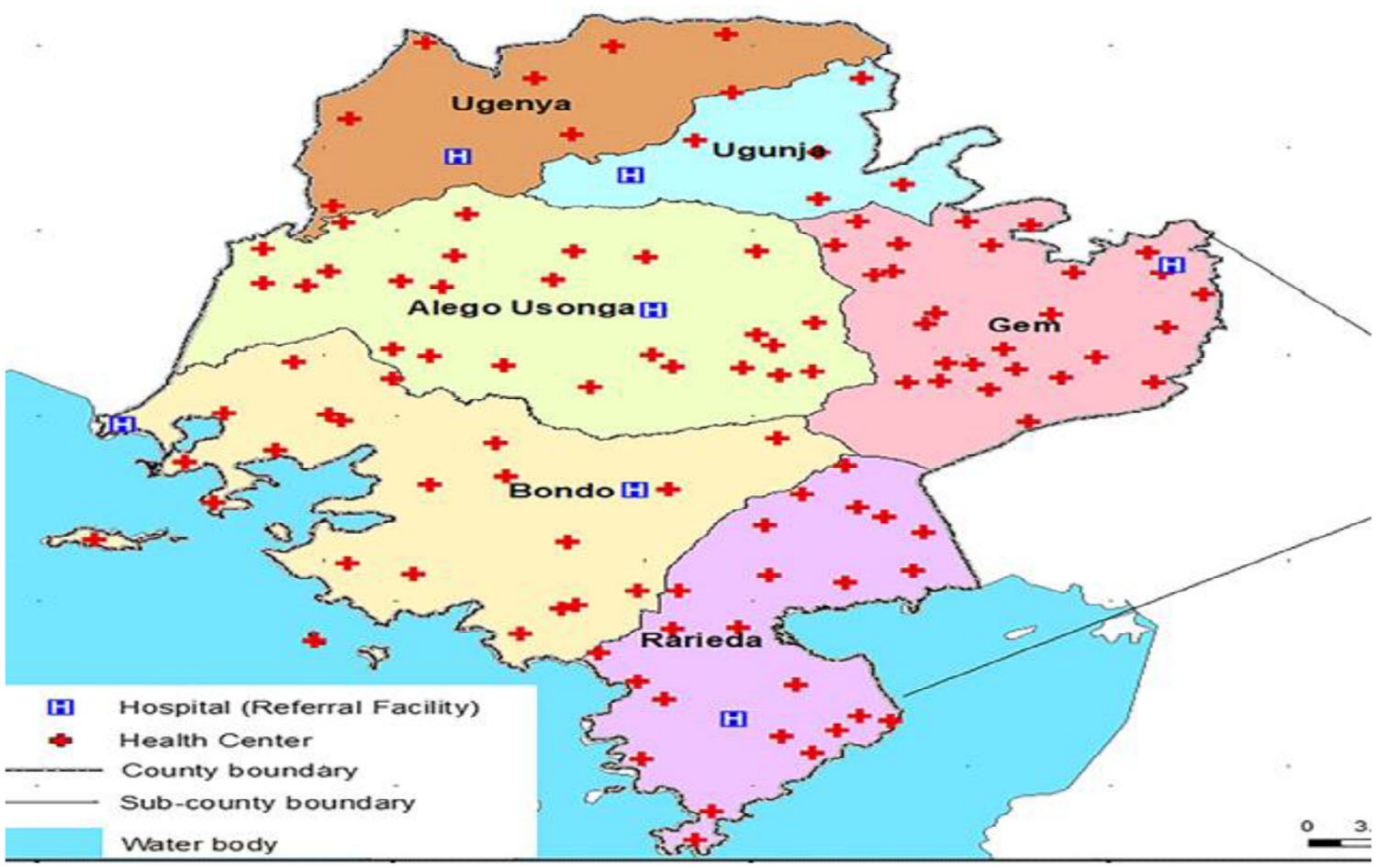
Siaya County in Kenya [17].

This study was conducted between May and July 2024 in Siaya County, located in Western Kenya (Figure 1). The county's economy relies mainly on agriculture, and it experiences a bimodal rainfall pattern with alternating wet and dry seasons and receives between 800 and 2200 mm of rainfall annually. These climatic conditions strongly influence agricultural productivity, seasonal food availability, and household food security [16]. Despite multiple development efforts, Siaya County continues to face challenges, including poverty, low agricultural productivity, high food insecurity, and a high burden of infectious diseases. These overlapping factors significantly affect the nutritional status of children under 5 years.

### 2.2 Study Population and Design

The study population comprised caregivers of children aged 0–60 months attending Siaya County Referral Hospital and food vendors operating in selected markets. Caregivers provided information on feeding practices, dietary quality, and household food availability, whereas vendors provided data on seasonal changes in food supply and price variations. A cross‐sectional study design incorporating both descriptive and inferential statistical analyses was used. A pilot study was conducted at Bondo Sub‐County Hospital among 10% (*n* = 11) of the sample size to pretest the tools and refine the questionnaires used in the main study. The study focused on identifying and evaluating locally available foods and dietary strategies to improve diversity during wet and dry seasons. Data collection was conducted using structured and unstructured questionnaires. A market survey was also conducted using random sampling to assess seasonal variations in food availability. The cross‐sectional design was chosen for its strength in estimating prevalence and exploring associations [18].

### 2.3 Inclusion Criteria

Participants were eligible if they met the following criteria:

- Caregivers of children aged 0–60 months residing within the study area.
- Children presenting at the facility with indicators of undernutrition (stunting, wasting, or underweight).
- Caregivers who provided informed consent.
- Food vendors operating within major markets in Siaya County and willing to participate.

### 2.4 Exclusion Criteria

Participants were excluded if

- The child was older than 5 years.
- The caregiver or child resided outside the study area.
- The child had medical conditions unrelated to nutrition, such as congenital abnormalities or chronic non‐nutrition‐related illnesses, as these could confound nutritional assessment. Verification was done using clinic records and caregiver reports.
- The caregiver or vendor declined to participate.

### 2.5 Sample Size Determination

The sample size was calculated using Cochran's formula suitable for populations greater than 10,000, *N* > 10,000 [19]:

$$N=\frac{Z^{2}P\left(1-P\right)}{D^{2}}$$

where *Z* = 1.96 (95% confidence level); *P* = 0.19 (prevalence of malnutrition from [6]); *D* = 0.05 (margin of error).

As the target population was less than 10,000 (202 caregivers visiting the nutritional clinic), the initial sample size was adjusted using the finite population correction (FPC) formula:

$$n_{f}=\frac{n}{1+\left(n/N\right)}=\frac{237}{1+\left(237/202\right)}=110$$

After adjustments and accounting for nonresponse, the final sample size was 110 participants. This sample size was deemed sufficient to detect meaningful differences in dietary diversity and food availability.

### 2.6 Sampling Technique

Purposive non‐probability and systematic probability sampling were utilized. The eligible children were those aged 0–60 months and those who met the inclusion criteria. The selection was done as follows:

#### 2.6.1 Selection of Hospital and Ward

Siaya County Referral Hospital was purposively selected, because it is one of the poorest counties in Kenya, and other problems within the community include low farm productivity, high rates of unemployment, and, most strikingly, resource‐constrained health facilities due to poverty. In addition to this, Siaya County also has one of the highest rates of morbidity and mortality in the country due to infectious diseases. Pediatric ward was purposively selected because this is where children aged 0–60 months are admitted.

#### 2.6.2 Selection of the Children From the Ward

In the ward, systematic sampling was utilized to select eligible children who met the inclusion criteria. Admission books in the pediatric ward were utilized. The interval was every second child in the admission books in the pediatric ward. The first two children in the register books and admission books in the ward were selected at random by balloting to determine the starting point. Data were collected until the required number of eligible (110) children in the ward was reached.

#### 2.6.3 Sampling Interval

To determine the interval for systematic sampling, the sample size was divided by the total population of children aged 0–60 months who met the inclusion criteria and were admitted at the pediatric ward.

#### 2.6.4 Sampling Frame

A sampling frame was used because all the units from the sampling frame could have an equal chance to be drawn and to occur in the sample. The sampling frame was all the children aged 0–60 months together with their caregivers in the study area and those whose caregivers gave consent for the study.

#### 2.6.5 Sampling Unit

The sampling unit included all caregivers with eligible children and caregivers who gave consent for the study.

### 2.7 Data Collection

Data collection employed both anthropometric and questionnaire‐based methods.

#### 2.7.1 Anthropometric Assessments

Measurements included

- **Weight** using calibrated Salter scales.
- **Length/height** using standardized measuring boards.
- **Mid‐upper arm circumference (MUAC)** using MUAC tapes. (All equipment underwent daily calibration, and measurements were taken twice by the same trained assessor to minimize interobserver variability.)

#### 2.7.2 Questionnaires and Market Surveys

Structured and unstructured questionnaires were used to gather data on:

- Demographic characteristics.
- Socioeconomic status.
- Dietary habits.
- Seasonal food availability.
- Food preservation practices.

Market vendors were sampled using simple random sampling, and data were collected on seasonal variations in the availability and price of key food items.

### 2.8 MDD Assessment

MDD was assessed using the [3] updated food groups, consisting of eight categories: breastmilk, grains, roots and tubers, legumes, nuts and seeds, dairy products, flesh foods, eggs, vitamin A–rich fruits and vegetables, and other fruits and vegetables.

Children who consumed foods from five or more food groups within the previous 24 h were classified as meeting MDD requirements.

### 2.9 Data Analysis

Data were analyzed using SPSS version 2016. Descriptive statistics summarized demographic data, anthropometric characteristics, and dietary patterns. WHO Anthro software generated standardized *Z*‐scores for height‐for‐age (HAZ), weight‐for‐age (WAZ), and weight‐for‐height (WHZ). The normality was assessed using the Shapiro–Wilk test, supported by visual inspection of histograms and Q–Q plots. Most continuous variables (age, weight, height, MUAC, and birth weight) followed a normal distribution (*p* > 0.05). Therefore, results were reported as mean ± SD, consistent with the WHO Child Growth Standards, which present anthropometric indices (HAZ, WAZ, and WHZ). This approach also supports parametric analyses, such as logistic regression, which identified predictors of dietary diversity and nutritional outcomes. For skewed variables, median and IQR were also checked, showing results consistent with mean ± SD. Chi‐square tests were used to assess associations between categorical variables [20, 21].

## 3 Results

### 3.1 Sociodemographic Characteristics of the Children

The sociodemographic and anthropometric characteristics of children aged 0–60 months in Siaya County, Kenya are presented in Table 1. Of these, 58.0% (*n* = 64) were female and 42.0% (*n* = 46) were male. The mean age was 16.7 months (SD = 12.6), with the majority 71.2% (*n* = 78), aged between 6 and 23 months, 18.9% (*n* = 21) between 24 and 59 months, and 9.9% (*n* = 11) under 6 months. Average birth weight was 3079.8 g (SD = 594.4), with 11.0% (*n* = 12) classified as low birth weight (<2500 g) and 89.0% (*n* = 98) within the normal range of 2500–4600 g. Household composition data indicated that 41.1% (*n* = 45) of children lived in households with one child under 5 years, 37.5% (*n* = 41) with two, 17.0% (*n* = 19) with three, and 4.5% (*n* = 5) with four or more children under 5. Nutritional assessment using *Z*‐score distributions showed notable deviation from standard growth metrics: 25.2% (*n* = 28) had *Z*‐scores of −4 SD, 14.4% (*n* = 16) at −3 SD, and 33.3% (*n* = 36) at −2 SD. 72.9% (*n* = 80) of children had scores below −2 SD. An additional 11.7% (*n* = 13) were at −1 SD, whereas 15.3% (*n* = 17) fell at 0 SD. Despite this, only 7.1% (*n* = 8) met the criteria for stunting, with 92.9% (*n* = 102) not classified as stunted. Anthropometric measures revealed a mean current weight of 9846.4 g (SD = 832.1). The average height of the children was 75.29 cm (SD = 56.1), and their average MUAC was 14.7 cm (SD = 20.9). These measurements showed significant variability as indicated by the relatively large standard deviations.

**TABLE 1:** Sociodemographic characteristics of children aged 0–60 months in Siaya County, Kenya.

Characteristic	Category	Frequency (*n*)	Percentage (%)
Gender of the child	Male	46	42.0
	Female	64	58.0
Age of the child in months	<6	11	9.9
	6–23	78	71.2
	24–59	21	18.9
Age of the child in months	Mean (SD)	—	16.7 (12.6)
Birth weight (g)	<2500	12	11.0
	2500–4600	98	89.0
Birth weight (g)	Mean (SD)	—	3079.8 (594.4)
Number of under 5 in the household	1	45	41.1
	2	41	37.5
	3	19	17.0
	4+	5	4.5
*Z*‐score	−4	28	25.2
	−3	16	14.4
	−2	36	33.3
	−1	13	11.7
	0	17	15.3
Stunting	No	102	92.9
	Yes	8	7.1
Current weight (g)	Mean (SD)	—	9846.4 (832.1)
Current height (cm)	Mean (SD)	—	75.29 (56.1)
MUAC (cm)	Mean (SD)	—	14.7 (20.9)

Abbreviation: MUAC, mid‐upper arm circumference.

### 3.2 Seasonal Household Food Availability

**FIGURE 2:**
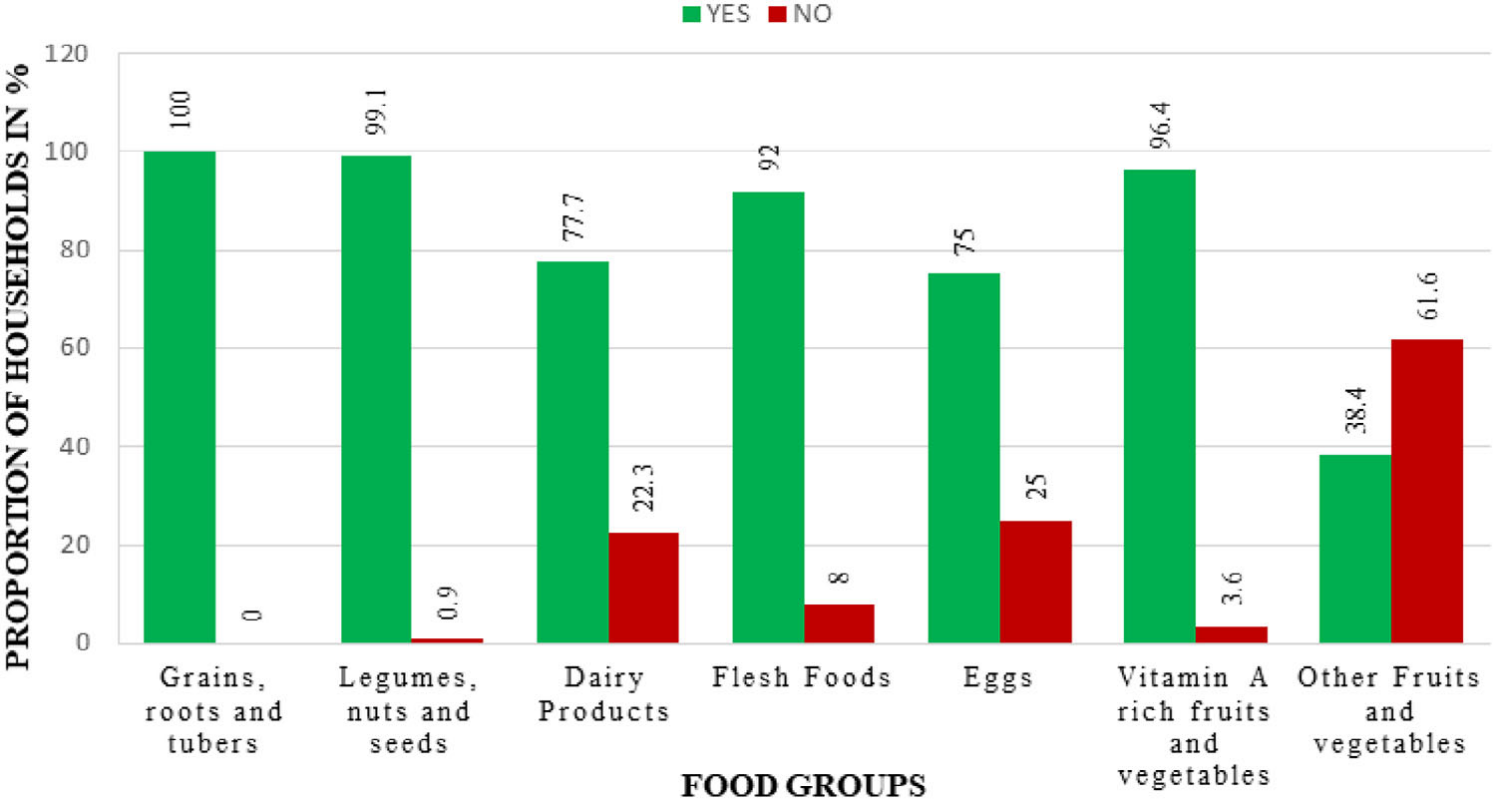
Seasonal distribution of household food group consumption.

Results for the household food group availability revealed distinct trends (Figure 2). Grains, roots, tubers, and plantains were universally available, with 100.0% (*n* = 110) of households reporting availability, showing widespread availability. Legumes and nuts exhibited near‐universal availability, with 99.1% (109) of households reporting access. Vitamin A–rich fruits and vegetables were accessible to 96.4% (*n* = 106) of households. Among animal‐source foods, flesh foods (meat, fish, and poultry) were available to 92.0% (101) of households, dairy products were available to 77.7% (*n* = 85), and eggs were available to 75.0% (*n* = 83). Other fruits and vegetables had the lowest reported availability, with 38.4% (*n* = 42) of households indicating access.

### 3.3 Household Consumption and Preservation of Foods

The results indicate that among the grains, roots, and tubers food group, maize had the highest fresh consumption of 90.2% (*n* = 101) and preservation rate of 51.8% (*n* = 58) across all food items (Table 2). Sweet potato, cassava, and Irish potato showed moderate fresh consumption of 62.5%–67.9% (*n* = 70–76) but lower preservation rates of 25.9%–28.6% (*n* = 29–32). Rice and millet were consumed fresh by 54.5%–69.6% (*n* = 61–78) of households, with preservation rates between 30.4% and 38.4% (*n* = 34–43). Yams had the lowest fresh consumption of 6.3% (*n* = 7) and preservation of 0.9% (*n* = 1) in this group. Among the legumes and nuts, beans recorded the highest fresh consumption 75.9% (*n* = 85) and preservation of 50.9% (*n* = 57), whereas groundnuts and green gram had moderate consumption and preservation rates. Peas, sesame, amaranth seeds, and lentils showed comparatively lower consumption and preservation. In the vitamin A–rich fruits and vegetables category, kales and other green leafy vegetables had the highest fresh consumption of 80.4% and 85.7% (*n* = 90 and 96) and preservation of 19.6% and 24.1% (*n* = 22 and 27). Mangoes and papayas also had substantial fresh consumption but lower preservation. Other vegetables, such as cabbage, exhibited moderate to low consumption and preservation rates. Oranges had the lowest consumption and preservation among other fruits, at 10.7% and 2.7% (*n* = 12 and 3), respectively. (“Rice was consumed fresh” means it was consumed cooked on the day of preparation and stored as dry grains; preservation methods beyond this were not applicable. Green leafy vegetables described in this study were amaranth leaves, cowpeas leaves, black nightshade, jute mallow, pumpkin leaves, spider plant.)

**TABLE 2:** Household consumption and preservation of selected foods.

Food group	Food item	Fresh consumption *n* (%)	Preservation *n* (%)
Grains, roots, and tubers	Maize	101 (90.2)	58 (51.8)
	Millet	61 (54.5)	34 (30.4)
	Rice	78 (69.6)	43 (38.4)
	Sorghum	47 (42.0)	28 (25.0)
	Sweet potato	76 (67.9)	32 (28.6)
	Cassava	75 (67.0)	29 (25.9)
	Irish potato	70 (62.5)	31 (27.7)
	Yams	7 (6.3)	1 (0.9)
Legumes, nuts, and seeds	Beans	85 (75.9)	57 (50.9)
	Peas	24 (21.4)	19 (17.0)
	Green grams	52 (46.4)	37 (33.0)
	Groundnut	52 (46.4)	30 (26.8)
	Sesame	10 (8.9)	10 (8.9)
	Amaranth seeds	7 (6.3)	2 (1.8)
	Lentils	0 (0.0)	1 (0.9)
Vitamin A–rich fruits and vegetables	Kales	90 (80.4)	22 (19.6)
	Green leafy vegetables	96 (85.7)	27 (24.1)
	Mango	80 (71.4)	20 (17.9)
	Papaya	59 (52.7)	17 (15.2)
	Spinach	45 (40.2)	9 (8.0)
	Carrot	19 (17.0)	6 (5.4)
	Pumpkin	34 (30.4)	10 (8.9)
Other fruits and vegetables	Cabbage	70 (62.5)	15 (13.4)
	Oranges	12 (10.7)	3 (2.7)

### 3.4 Minimum Dietary Diversity

Among children who met the MDD criteria, maize was the predominant staple, consumed by 95.2% (*n* = 20) (Table 3). Other grains and tubers, including rice at 47.6% (*n* = 10), millet at 42.9% (*n* = 9), and Irish potato at 42.9% (*n* = 9), were moderately consumed, whereas sorghum at 23.8% (*n* = 5) and sweet potato at 28.6% (*n* = 6) had lower consumption rates. In the legumes and nuts category, beans, peas, and groundnuts were each consumed by 33.3% (*n* = 7) of children, whereas amaranth seeds were less common at 23.8% (*n* = 5). Dairy consumption varied: Fresh milk was consumed by over half of the children (52.4% (*n* = 11)), whereas infant formula (28.6% (*n* = 6)) and other dairy products (33.3% (*n* = 7)) were less frequently reported. Flesh food intake showed clear variation with fish being the most commonly consumed animal‐source food with 66.7% (*n* = 14). Chicken meat and pork were each reported by 23.8% (*n* = 5) of children, whereas beef had the lowest consumption of 14.3% (*n* = 3). Chicken eggs were consumed at 38.1% (*n* = 8). Vitamin A–rich foods were consumed at moderate levels, with 42.9% (*n* = 9) of children reporting intake of other vitamin A–rich foods and 28.6% (*n* = 6) consuming kales.

**TABLE 3:** Local foods consumed by children meeting minimum dietary diversity (MDD).

Food group	Food item	Yes (%)	No (%)
Grains, roots, and tubers	Maize	20 (95.2)	1 (4.8)
	Millet	9 (42.9)	12 (57.1)
	Rice	10 (47.6)	11 (52.4)
	Sorghum	5 (23.8)	16 (76.2)
	Sweet potato	6 (28.6)	15 (71.4)
	Irish potato	9 (42.9)	12 (57.1)
Legumes, nuts, and seeds	Beans	7 (33.3)	14 (66.7)
	Peas	7 (33.3)	14 (66.7)
	Groundnut	7 (33.3)	14 (66.7)
	Amaranth seeds	5 (23.8)	16 (76.2)
Dairy products	Fresh milk	11 (52.4)	10 (47.6)
	Infant formula	6 (28.6)	15 (71.4)
	Other dairy products	7 (33.3)	14 (66.7)
Flesh foods	Chicken meat	5 (23.8)	16 (76.2)
	Beef	3 (14.3)	18 (85.7)
	Pork	5 (23.8)	16 (76.2)
	Fish	14 (66.7)	7 (33.3)
Eggs	Chicken egg	8 (38.1)	13 (61.9)
Vitamin A–rich foods	Kales	6 (28.6)	15 (71.4)
	Other vitamin A–foods	9 (42.9)	12 (57.1)

**FIGURE 3:**
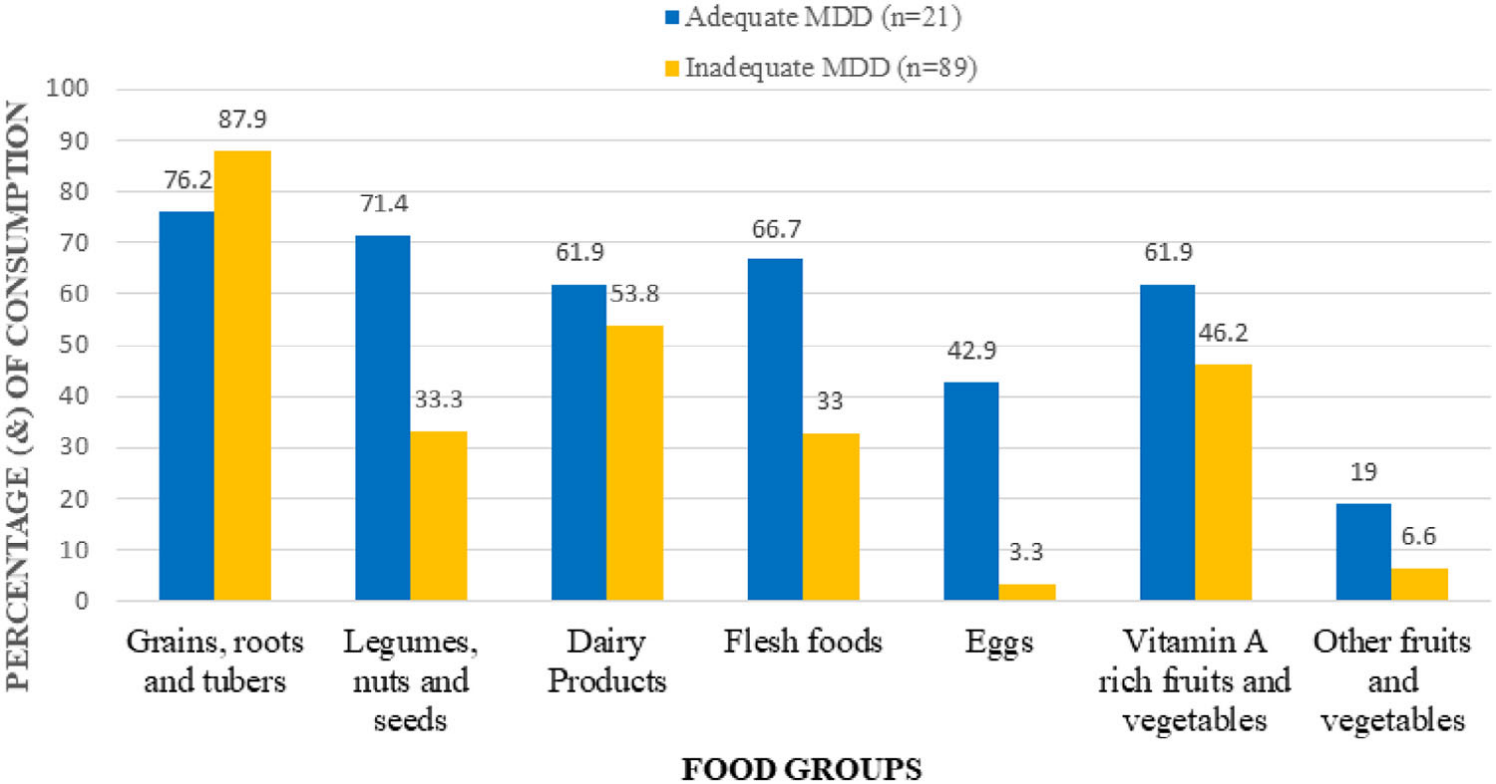
Food group consumption based on dietary diversity. MDD, minimum dietary diversity.

Children who met the MDD threshold consumed foods from all seven groups more frequently than those who did not (Figure 3). The most commonly consumed group was grains, roots, and tubers in 76.2% (*n* = 16) of children with adequate MDD and 87.9% (*n* = 78) of those with inadequate MDD. Legumes and nuts were reported in the diets of 71.4% (*n* = 15) of the adequate group compared to 34.1% (*n* = 30) of the inadequate group (*p* value = 0.0017). Dairy products were consumed by 61.9% (*n* = 13) of children meeting MDD compared to 53.8% (*n* = 48) MDD‐inadequate children. There was a significant difference in proportion flesh consumption between children who had adequate MDD (66.7% (*n* = 14)) and those with inadequate MDD (33% (*n* = 29)) (*p* value = 0.0044). The greatest difference was observed in egg consumption where 42.9% (*n* = 9) of MDD‐adequate children ate eggs compared to 3.3% (*n* = 3) of MDD‐inadequate children. Vitamin A–rich fruits and vegetables were consumed by 61.9% (*n* = 13) of children meeting MDD compared to 46.2% (*n* = 41) MDD‐inadequate children. Other fruits and vegetables were the least consumed food group, reported in only 19% (*n* = 4) of children meeting the MDD threshold and 6.6% (*n* = 6) among those with inadequate dietary diversity. (Differences in consumption between children who met and did not meet the MDD threshold were assessed using Chi‐square tests of proportions, with significant differences observed (*p* < 0.001).)

#### 3.4.1 MDD Proportion

**FIGURE 4:**
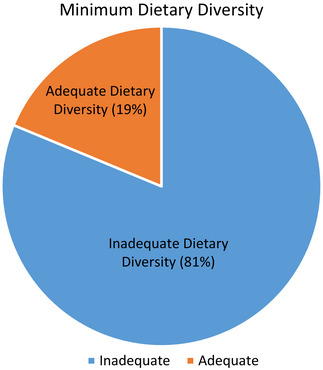
MDD proportion.

As shown in Figure 4, the MDD proportion, determined on the basis of a 24 h recall method, meeting the WHO threshold (≥5 food groups), showed that 21 (19%, 95% CI: 12.5–27.2) of the children had achieved MDD. (Exclusive breastfeeding for infants under 6 months was not assessed, since the MDD indicator applies only to children aged 6–23 months who consume complementary foods.)

## 4 Discussion

The sociodemographic and anthropometric characteristics of children under 5 years old in Siaya County indicate significant nutritional vulnerabilities. A large proportion of these children are between 6 and 23 months of age. This is a critical developmental stage marked by rapid growth and increased risk of nutrient deficiencies due to elevated nutritional demands [22]. The mean birth weight was within normal limits. However, 11% (*n* = 12) of children were born with low birth weight. This suggests possible maternal health challenges, including poor nutrition, anemia, and inadequate antenatal care. Recent studies confirm that maternal anemia, depression, and underweight status significantly raise the risk of low birth weight [23, 24]. Although stunting affected 7.1% (*n* = 8) of children, almost three‐quarters had lower HAZ *Z*‐scores indicating widespread growth faltering. This highlights the limitations of binary stunting measures and supports using full *Z*‐score distributions for more sensitive detection of chronic undernutrition [25, 26]. MUAC measurements showed high variability, possibly due to true differences in nutritional status or inconsistent measurement techniques. Although repeated measurements by the same observer are reliable, interobserver variability remains a concern, particularly in community settings [27, 28].

As this was a cross‐sectional study, the reported high proportions of households accessing staple and protein foods reflect availability at the time of data collection, rather than consistent access over time. However, availability of nutrient‐dense foods, such as fruits, vegetables, dairy products, and eggs, remains limited. Dietary imbalance contributes to insufficient intake of critical micronutrients that are vital for fetal development, immune competence, and linear growth in children [29]. Despite widespread consumption of staples and pulses, dietary diversity remains suboptimal. Intake of non‐vitamin A–rich fruits and vegetables is low, and consumption of animal‐source foods is inconsistent. This is has been attributed to seasonal variability, limited market access, and sociocultural factors that reduce diet quality and nutritional adequacy [30]. Similar studies have revealed consistent dietary patterns characterized by heavy reliance on staple crops and limited intake of diverse, nutrient‐rich foods. A previous study conducted in Ugenya revealed that households heavily rely on maize, beans, and cassava, while consuming few fruits and vegetables, contributing to undernutrition [31]. Similarly, an earlier study in Rarieda highlighted that cultural norms, seasonal food preferences, and gender roles significantly influence food choices often restricting access to micronutrient‐rich foods [30]. Comparable trends have been observed across East Africa, where structural barriers continue to limit dietary diversity at the household level [29, 32]. In Kenya, food insecurity remains a persistent challenge. According to KIPPRA [29], only 22.3% of Kenyan households are classified as food secure, with rural populations disproportionately affected. Agricultural interventions such as irrigation and improved seed varieties have enhanced food production. However, access remains inequitable due to gender‐based resource disparities and limited distribution infrastructure. Structural factors such as land tenure, livestock ownership, and market access influence food security in Sub‐Saharan Africa. These factors play a key role in determining household food availability and access. Moreover, climate variability, economic shocks, and global supply disruptions have increased child malnutrition and worsened nutritional vulnerabilities [33, 34].

Household‐level food availability and consumption are significantly influenced by postharvest preservation practices. Maize and beans are the main dietary staples, consumed both in fresh form and preserved for future use. Maize, the leading crop, is traditionally stored in granaries or sacks, but losses of 21%–36% occur due to pests, moisture, and poor handling [35]. The adoption of hermetic storage, such as PICS bags, has cut losses to below 1%, preserved grain quality, and strengthened household food security [36, 37, 38]. Roots and tubers, such as sweet potato, cassava, and Irish potato, are important components of local diets. However, they are rarely preserved due to their high perishability and the lack of suitable storage facilities. Under normal storage conditions, sweet potatoes have a postharvest shelf life of only 4–7 days. This short storage period limits their availability during scarcity unless they are processed through drying or fermentation [39]. Vitamin A–rich leafy vegetables, including kale, cowpea leaves, and amaranth, are primarily consumed fresh. They are rarely preserved due to their delicate structure and high susceptibility to spoilage. Traditional preservation methods, including sun drying and fermentation, are still widely used. However, the uptake of improved technologies is hindered by economic constraints, limited extension support, and inadequate infrastructure [40]. Pulses are well suited to preservation through sun drying, which extends their shelf life and supports year‐round availability. Pulses provide key nutrients, and their preservation is essential for dietary adequacy in resource‐limited settings [41]. Limited preservation of nutrient‐rich foods like fruits and vegetables causes seasonal dietary shortages. Increasing the use of advanced preservation techniques, including solar drying, safe storage systems, and community training, has the potential to enhance dietary diversity. Such methods could also help ensure more consistent year‐round access to essential nutrients [42]. Preservation practices are a strategic means of reducing seasonal food insecurity and strengthening household resilience to fluctuations in food supply.

Among children under 5 who met the MDD threshold, maize was the predominant staple, consumed by 95.2%. In contrast, intake of other staples, such as sorghum and sweet potato, was considerably lower. Previous studies show that cereals like maize provide important sources of energy. However, when consumed alone, they lack sufficient nutrients and can increase the risk of micronutrient deficiencies if dietary diversity is limited [43]. It is also evident that in Western Kenya, although staples like sorghum are available, cultural preferences especially during hunger seasons favor maize limiting dietary diversity. Only about one‐third of the children consumed pulses, which is modest considering their affordability and nutritional benefits as sources of protein and micronutrients. Similar studies elsewhere show that legume consumption among young children is below recommended levels thus limiting dietary diversity and nutrient intake. Mekonen [44] found low legume consumption in Ethiopian children aged 6–23 months, highlighting the need to promote legumes to improve diet quality in the region. Animal‐source food consumption varied among children at the time of the study, with eggs more commonly consumed among those meeting the MDD threshold; however, these findings reflect cross‐sectional intake rather than longitudinal patterns. Intervention studies have shown that giving one egg per day significantly improves the chances of meeting MDD thresholds, although effects on linear growth may be limited [45]. Other studies show that eating animal‐source foods improves growth and lowers stunting risk more effectively than plant‐based foods alone [46]. Animal‐source foods in Sub‐Saharan Africa effectively improve growth and reduce stunting compared to plant‐based foods [47]. Fruit and vegetable consumption was notably low, with few children meeting the MDD consuming them and none among those with inadequate MDD. Despite local production, fruit and vegetable intake in East Africa remains far below recommended levels [48]. For example, a Kenyan survey reported that 95% of women consumed inadequate amounts of fruits and vegetables, a pattern also observed in national data for the adult population [49]. This study adopted cross‐sectional design that lacks the capacity to capture temporal relationships and increased the likelihood of noncausal associations. The study has been done in a single site with limited environmental and sociodemographic diversity making it difficult to generalize the findings in heterogeneous populations. In addition, the participant's selection based on predefined criteria may have introduced selection bias affecting representativeness of the findings.

## 5 Conclusions

This study highlights the significant dietary and nutritional vulnerabilities among children under 5 years in Siaya County. Dietary diversity was generally low, with only a small proportion of children meeting the WHO‐recommended MDD. Consumption of nutrient‐dense foods, such as pulses, eggs, and flesh foods, was limited, largely due to seasonal fluctuations in availability and affordability. These challenges were compounded by socioeconomic constraints and the effects of climate variability on local food systems. Traditional food preservation methods, including sun drying and smoking, remain important strategies for extending food availability during periods of scarcity. However, their potential to support dietary diversity could be strengthened through improved training on safe and nutrient‐preserving practices. Overall, the findings underscore the need for integrated, context‐specific interventions that enhance dietary quality among young children. Efforts to improve nutrition outcomes should focus on strengthening household food security, promoting consumption of locally available nutrient‐dense foods, and increasing caregiver awareness of optimal feeding practices. Enhancing climate‐resilient agricultural practices and supporting community‐based nutrition programs could further contribute to improving the nutritional status of children in the county.

## Author Contributions

**Gladwel Kuya Sikoyo**: conceptualization, formal analysis, methodology, project administration, validation, visualization, writing – original draft, writing – review and editing. **Judith Mangeni**: conceptualization, project administration, funding acquisition, methodology, resources, supervision, visualization, writing – original draft, writing – review and editing. **George Ayodo**: conceptualization, funding acquisition, methodology, resources, supervision, visualization, writing – original draft, writing – review and editing. **Fanuel Kawaka**: supervision, visualization, writing – original draft, writing – review and editing. **Diana Menya**: project administration, supervision, validation writing – review and editing. **Oscar Kambona**: supervision, validation, methodology, writing – review and editing. **Antony Ochung**: methodology, analysis, writing – review and editing.

## Funding

This work was partially funded by the Climate Adaptation Research Program, which is made possible by the generous support of the American people through the USAID Bureau for Humanitarian Assistance (Award# 720FDA20CA00006). The USAID administers the US foreign assistance program providing economic and humanitarian assistance in more than 80 countries worldwide. The Climate Adaptation Research Program in Africa is implemented by the Partners for Enhancing Resilience for People Exposed to Risks (PERIPERI‐U) Network in the Centre for Collaboration in Africa at Stellenbosch University and the Humanitarian Assistance Technical Support initiative in the Bureau of Applied Research in Anthropology at the University of Arizona USA.

## Ethics Statement

The study was approved by the Jaramogi Oginga Odinga University of Science and Technology (JOOUST) Ethics Review Committee (ERC No. ERC 41/2/24‐05) and licensed by the National Commission for Science, Technology and Innovation (NACOSTI)—No. NACOSTI/P/24/33789. Final approval was granted by the County Health Management Team of the Siaya County Government.

## Consent

Caregivers and mothers at the pediatric clinic were made aware of the study's objectives and the nature of their participation. Verbal consent was obtained at first contact with the study participant, and written consent was signed after taking through every participant on the critical elements of the study. Participants were assured that their decision to participate was voluntary and would neither affect their relationship with their health providers nor the care they receive from them. Participants remained anonymous, and the information provided was stored confidentially. Each participant was assigned a random unique identifier, and they were assured of the confidentiality of their data.

## Conflicts of Interest

The authors declare no conflicts of interest.

## Clinical Trial Registration

This study involved interviews with human participants but did not comprise a clinical trial.

## Permission to Reproduce Material From Other Sources

All third‐party material included in this manuscript is used with the necessary permissions.

## Data Availability

Data will be available upon publication in a repository.

## References

[1] Z. Khan and A. Ali , “Global Food Insecurity and Its Association With Malnutrition,” Emerging Challenges in Agriculture and Food Science 8 (2023): 2–19.

[2] WHO , The State of Food Security and Nutrition in the World 2020: Transforming Food Systems for Affordable Healthy Diets (Food and Agriculture Organization, 2020).

[3] WHO/UNICEF , Indicators for Assessing Infant and Young Child Feeding Practices (World Health Organization, 2023).

[4] P. Codjia , L. Kiige , C. Rudert , et al., “Improving Complementary Feeding Practices, Programs and Policies for Optimal Early Childhood Nutrition in Kenya: What Would Work?,” Maternal & Child Nutrition 20 (2024): e13616. 38204287 10.1111/mcn.13616 PMC10782134

[5] H. A. Ewune , R. K. Abebe , D. Sisay , and G. A. Tesfa , “Prevalence of Wasting and Associated Factors Among Children Aged 2–5 Years, Southern Ethiopia: A Community‐Based Cross‐Sectional Study,” BMC Nutrition 8 (2022): 160. 36585708 10.1186/s40795-022-00657-x PMC9805277

[6] KDHS , Kenya Demographic and Health Survey. Key Indicators Report (KDHS, 2022).

[7] FAO , The State of Food Security and Nutrition in the World 2023: Urbanization, Agrifood Systems Transformation and Healthy Diets across the Rural–Urban Continuum (Food and Agriculture Organization of the United Nations, 2023).

[8] A. Rossati , “Global Warming and Its Health Impact,” International Journal of Occupational and Environmental Medicine 8 (2016): 7–20. 10.15171/ijoem.2017.963 PMC6679631 28051192

[9] A. P. B. Núñez , I. Gutiérrez‐Montes , H. E. Hernández‐Núñez , et al., “Diverse Farmer Livelihoods Increase Resilience to Climate Variability in Southern Colombia,” Land Use Policy 131 (2023): 106731.

[10] E. A. Anyona , J. Obuoyo , and I. Mutavi , “Influence of Home Gardening on Household Food Security in Rarieda Sub‐County of Siaya County in Kenya,” International Journal of Research and Innovation in Social Science, International Journal of Research and Innovation in Social Science (IJRISS) 8, no. 6 (2024): 111–128.

[11] S. J. Osendarp , H. Martinez , G. S. Garrett , et al., “Large‐Scale Food Fortification and Biofortification in Low‐and Middle‐Income Countries: A Review of Programs, Trends, Challenges, and Evidence Gaps,” Food and Nutrition Bulletin 39 (2018): 315–331. 29793357 10.1177/0379572118774229 PMC7473077

[12] A. Shehzad , H. A. Suleria , and S. Akram , “Nutritional Interventions for Tackling Micronutrient Deficiencies,” Frontiers Media SA 11 (2024): 1451493. 10.3389/fnut.2024.1451493 PMC11446884 39364154

[13] N. Chanza and W. Musakwa , “Revitalizing Indigenous Ways of Maintaining Food Security in a Changing Climate: Review of the Evidence Base From Africa,” International Journal of Climate Change Strategies and Management 14 (2022): 252–271.

[14] P. Codjia , E. Kutondo , P. Kamudoni , et al., “Mid‐Term Evaluation of Maternal and Child Nutrition Programme (MCNP II) in Kenya,” BMC Public Health [Electronic Resource] 22 (2022): 2191. 36443721 10.1186/s12889-022-14627-2 PMC9702643

[15] L. M. Waswa , I. Jordan , M. B. Krawinkel , and G. B. Keding , “Seasonal Variations in Dietary Diversity and Nutrient Intakes of Women and Their Children (6–23 Months) in Western Kenya,” Frontiers in Nutrition 8 (2021): 636872. 33763444 10.3389/fnut.2021.636872 PMC7982591

[16] County Government of Siaya , Siaya County Climate Change Action Plan 2023–2028 (Department of Water, Sanitation, Environment, Natural Resources and Climate Change, The County Government of Siaya, 2023).

[17] J. J. O. Ouko , M. K. Gachari , A. W. Sichangi , and V. Alegana , “Geographic Information System‐Based Evaluation of Spatial Accessibility to Maternal Health Facilities in Siaya County, Kenya,” Geographical Research 57 (2019): 286–298.

[18] X. Wang and Z. Cheng , “Cross‐Sectional Studies: Strengths, Weaknesses, and Recommendations,” Chest 158 (2020): S65–S71. 32658654 10.1016/j.chest.2020.03.012

[19] W. G. Cochran , Sampling Techniques (John Wiley & Sons, 1977).

[20] WHO , “WHO Child Growth Standards Based on Length/Height, Weight and Age,” Acta Paediatrica 95 (2006): 76–85. 10.1111/j.1651-2227.2006.tb02378.x 16817681

[21] WHO , Recommendations for Data Collection, Analysis and Reporting on Anthropometric Indicators in Children under 5 Years Old (WHO, 2019).

[22] G. W. Kihagi , L.‐S. Hansen , E. Agure , et al., “‘Counselling Is Not Just Providing Information’: Perceptions of Caregivers and Stakeholders on the Design of Nutrition and Health Counselling Interventions for Families With Young Children in Rural Kenya,” BMC Health Services Research 24 (2024): 597. 38715044 10.1186/s12913-024-10872-w PMC11077832

[23] H. Arabzadeh , A. Doosti‐Irani , S. Kamkari , M. Farhadian , E. Elyasi , and Y. Mohammadi , “The Maternal Factors Associated With Infant Low Birth Weight: An Umbrella Review,” BMC Pregnancy and Childbirth 24 (2024): 316. 38664680 10.1186/s12884-024-06487-y PMC11044292

[24] N. Chahal , T. Qureshi , S. Eljamri , J. M. Catov , and P. K. Fazeli , “Impact of Low Maternal Weight on Pregnancy and Neonatal Outcomes,” Journal of the Endocrine Society 9 (2025): bvae206. 10.1210/jendso/bvae206 PMC11635456 39669656

[25] P. A. Chanyarungrojn , N. Lelijveld , A. Crampin , et al., “Tools for Assessing Child and Adolescent Stunting: Lookup Tables, Growth Charts and a Novel Appropriate‐Technology “MEIRU” Wallchart—A Diagnostic Accuracy Study,” PLOS Global Public Health 3 (2023): e0001592. 37450437 10.1371/journal.pgph.0001592 PMC10348557

[26] M. Ogero , N. Scott , V. Kirogo , and A. K. Ngugi , “Improving Nutrition Outcomes Through Enhanced Allocative Efficiency of Investments in 24 High Risk Counties in Kenya: An Optima Nutrition Modelling Study,” PLoS ONE 20 (2025): e0323391. 40424473 10.1371/journal.pone.0323391 PMC12112240

[27] H. A. Saeed , J. B. Mogendi , R. Akparibo , and P. Kolsteren , “Reliability of Mid‐Upper Arm Circumference Measurements Taken by Community Health Nurses,” Current Research in Nutrition and Food Science Journal 3 (2015): 26–35.

[28] E. Sendaula , J. Kalyango , N. B. Nabukeera , and C. Karamagi , “Performance of MUAC and Associated Factors in the Prediction of Acute Malnutrition Among Children 6–59 Months at Mulago Hospital, Kampala,” Journal of Nutrition and Human Health 3, no. 2 (2020): 1–7.

[29] KIPPRA , Status of Food and Nutrition Security in Kenya: An Implementation of the Framework for Harmonizing Nutrition Indicators (Kenya Institute for Public Policy Research and Analysis, 2024).

[30] M. M. Musyoka , Socio‐cultural Drivers of Foodways and Their Implication on Household Food Security in Rarieda Sub‐County, Siaya County (Uon, 2021).

[31] E. O. Odinga , D. Mamboleo , and D. Nyantika , “Assessing the Contributions and Quality of Various Food Crops Harvested by Household on Food Security in Ugenya Sub‐County, Siaya County, Kenya,” Sciences 3 (2022): 14.

[32] H. A. Paulo , J. Andrew , P. Luoga , et al., “Minimum Dietary Diversity Behaviour Among Children Aged 6 to 24 Months and Their Determinants: Insights From 31 Sub‐Saharan African (SSA) Countries,” BMC Nutrition 10 (2024): 160. 39695838 10.1186/s40795-024-00967-2 PMC11656542

[33] M. B. Rother , M. S. Sosa , and M. D. Kim , Tackling the Global Food Crisis: Impact, Policy Response, and the Role of the IMF (International Monetary Fund, 2022).

[34] M. Toure , Agricultural Adaptation to Climate Change in Sub‐Saharan Africa (Springer, 2025).

[35] H. De Groote , F. N. Muteti , and A. Y. Bruce , “On‐Farm Storage Loss Estimates of Maize in Kenya Using Community Survey Methods,” Journal of Stored Products Research 102 (2023): 102107. 37361490 10.1016/j.jspr.2023.102107 PMC10285508

[36] T. Deressa , D. Diro , G. Getachew , and G. Demissie , “Performance Evaluation of Metal Silo and PICS Bag for Maize Grain Storage Against Insect Pest Infestation and Grain Quality,” EAS Journal of Nutrition and Food Sciences 7 (2025): 1–9.

[37] T. Tubbs , D. Baributsa , and C. Woloshuk , “Impact of Opening Hermetic Storage Bags on Grain Quality, Fungal Growth and Aflatoxin Accumulation,” Journal of Stored Products Research 69 (2016): 276–281. 27990032 10.1016/j.jspr.2016.10.003 PMC5146292

[38] H. Zacharia , S. Feleke , M. Nyaa , and D. Baributsa , “Adoption and Impacts of Hermetic Storage on Household Food Insecurity Among Maize Producers' Organizations in Tanzania,” Discover Agriculture 2 (2024): 98.

[39] P. E. Abidin , J. Kazembe , R. A. Atuna , et al., “Sand Storage, Extending the Shelf‐Life of Fresh Sweetpotato Roots for Home Consumption and Market Sales,” Journal of Food Science and Engineering 6 (2016): 227–236.

[40] H. Xu , M. Lei , J. Li , et al., “Effects of Different Drying Methods on the Physicochemical and Functional Properties of *Pyracantha fortuneana* (Maxim.) Li Fruit,” LWT 187 (2023): 115383.

[41] R. M. Ariong , D. M. Okello , M. H. Otim , and P. Paparu , “The Cost of Inadequate Postharvest Management of Pulse Grain: Farmer Losses Due to Handling and Storage Practices in Uganda,” Agriculture & Food Security 12 (2023): 20.

[42] H. M. Lisboa , M. B. Pasquali , A. I. Dos Anjos , et al., “Innovative and Sustainable Food Preservation Techniques: Enhancing Food Quality, Safety, and Environmental Sustainability,” Sustainability 16 (2024): 8223.

[43] Y. J. H. Galani and C. Orfila , “A Review of Micronutrient Deficiencies and Analysis of Maize Contribution to Nutrient Requirements of Women and Children in Eastern and Southern Africa,” Critical Reviews in Food Science and Nutrition 62 (2022): 1568–1591. 33176441 10.1080/10408398.2020.1844636

[44] E. G. Mekonen , “Animal Source Food Consumption and Its Determinants Among Children Aged 6 to 23 Months in Sub‐Saharan African Countries: A Multilevel Analysis of Demographic and Health Survey,” BMC Public Health [Electronic Resource] 24 (2024): 2060. 39085814 10.1186/s12889-024-19628-x PMC11290212

[45] C. K. Lutter , B. L. Caswell , C. D. Arnold , et al., “Impacts of an Egg Complementary Feeding Trial on Energy Intake and Dietary Diversity in Malawi,” Maternal & Child Nutrition 17 (2021): e13055. 33128502 10.1111/mcn.13055 PMC7729770

[46] K. F. Michaelsen , C. Hoppe , N. Roos , et al., “Choice of Foods and Ingredients for Moderately Malnourished Children 6 Months to 5 Years of Age,” Food and Nutrition Bulletin 30 (2009): S343–S404. 19998864 10.1177/15648265090303S303

[47] M. A. Zemene , N. Kebede , R. M. Anteneh , et al., “Determinants of Animal Source Food Consumption Among Children Aged 6–23 Months in Sub‐Saharan Africa: Multilevel Mixed Effect Model,” Scientific Reports 14 (2024): 26294. 39487171 10.1038/s41598-024-73840-8 PMC11530564

[48] J. Sarfo , E. Pawelzik , and G. B. Keding , “Fruit and Vegetable Processing and Consumption: Knowledge, Attitude, and Practices Among Rural Women in East Africa,” Food Security 15 (2023): 711–729.

[49] S. Pengpid and K. Peltzer , “The Prevalence and Social Determinants of Fruit and Vegetable Consumption Among Adults in Kenya: A Cross‐Sectional National Population‐Based Survey, 2015,” Pan African Medical Journal 31 (2018): 137. 31037197 10.11604/pamj.2018.31.137.17039 PMC6462356

